# The Biomechanics of Converting Torque into Spin Rate in Spin Bowlers Analyzed with a Smart Cricket Ball

**DOI:** 10.1007/s43465-023-00943-1

**Published:** 2023-07-10

**Authors:** Franz Konstantin Fuss, Batdelger Doljin, René E. D. Ferdinands

**Affiliations:** 1 Chair of Biomechanics, Faculty of Engineering Science, University of Bayreuth, 95440 Bayreuth, Germany; 2 Smart Products Engineering Program, Swinburne University, Melbourne, VIC 3000 Australia; 3 Discipline of Exercise and Sports Science, School of Health Sciences, Faculty of Medicine and Health, University of Sydney, Sydney, NSW 2141 Australia

**Keywords:** Cricket, Smart ball, Biomechanics, Spin rate, Torque, Skill parameters, Profiling, Talent identification

## Abstract

**Background:**

In previous studies that analyzed the biomechanics of spin bowling with a smart cricket ball, it was evident that not all the torque applied to the ball was converted into spin rate, with varying losses among different bowlers. This study aims to investigate the factors contributing to these losses.

**Methods:**

Developed in 2011, the world's first intelligent cricket ball features five physical and five skill performance parameters. Our study correlates five skill parameters with the ratio of total torque to spin rate to determine the most influential skill parameter.

**Results:**

The parameter that most affected the conversion of torque to spin rate was the ratio of maximum angular acceleration to maximum angular velocity. Since the unit of the latter ratio is measured in s^–1^ or Hz, we hypothesized that the duration of a time-window in which the torque is generated could be a factor in determining the effectiveness of torque to spin conversion. Upon closer examination of the data, we discovered that the spin torque (the torque component that boosts the spin rate) generated earlier in relation to the release point led to greater conversion of total torque into spin rate. Paradoxically, this occurred at smaller peak spin torques. As the time-window of the spin torque widens despite its decreasing magnitude, the angular impulse increases.

**Conclusions:**

As the skill parameter calculated from the ratio of maximum angular acceleration to maximum angular velocity correlates well with the time-window during which torque is generated, it can serve as a good indicator of skillful torque to spin conversion, and a potential parameter for talent identification.

## Introduction

Fast spin rate is a critical performance outcome in spin bowling [1]. Spin bowlers impart spin to the cricket ball by applying torque to the ball via an intricate motion of the fingers which are arranged spatially around the circumference of the ball. There are many different types of spin bowling deliveries, but they can generally be divided into two main categories: finger spin and wrist spin. Since the advent of the world’s first smart cricket ball in 2011 [2, 3], the relationships between this torque and spin rate can be quantified for these types of deliveries. From a purely mechanical perspective, the conversion of torque into angular velocity is explained by the following equation:

1 $$\omega = {\int }_{{t}_{1}}^{{t}_{2}}\alpha \ \mathrm{d}t={I}^{-1}{\int }_{{t}_{1}}^{{t}_{2}}T \ \mathrm{d}t,$$

where *ω* denotes the angular velocity (spin rate); *α* denotes the angular acceleration; *t* is the time; *t*_1_, *t*_2_ are the boundaries of the integration window; *T* is the torque imparted to the ball by the bowler; and *I* is the moment of inertia of the ball.

**Fig. 1:**
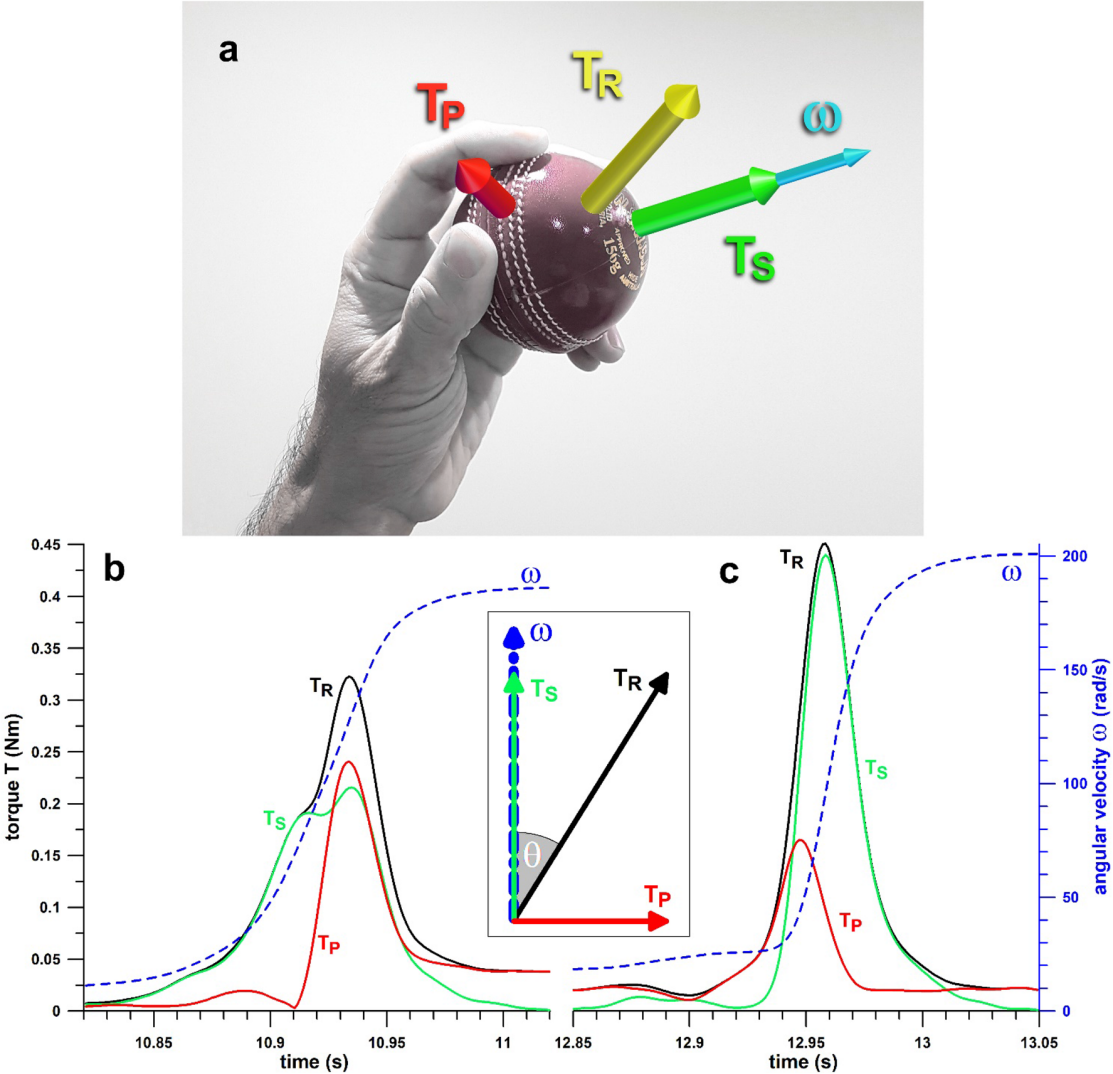
Principle of the relationship of torque and angular velocity vectors; **a** resultant torque vector and its components and their relationship to the angular velocity vector for a ball to be released with a wrist spin (lefthanded bowler); **b**, **c** torques and angular velocity versus time graphs of two bowlers; **b** wrist spinner, **c** finger spinner; *T*_R_: resultant torque, *T*_S_: spin torque, *T*_P_: precession torque, *ω*: angular velocity; the release points are located at the end of each time axis (11.02 s and 13.05 s); inset: *T*_R_ vector and its components *T*_S_ and *T*_P_, *θ* denotes the included angle between *T*_R_ and *ω*

A mechanical problem arises when the **T**_**R**_ (resultant torque) vector imparted to the ball does not align with the *ω* vector [4] (Fig. 1). The included angle *θ* between **T**_**R**_ and *ω* divides **T**_**R**_ into two components (Fig. 1), the spin torque **T**_**S**_ parallel to *ω*, and the precession torque **T**_**P**_ perpendicular to *ω* [5]. However, only **T**_**S**_ changes the magnitude of *ω*, whereas **T**_**P**_ forces the *ω* vector to move into the **T**_**R**_ vector without affecting the magnitude of *ω* [5]. Therefore, **T**_**P**_ can be considered a wasted or lost torque, incapable of increasing the spin rate of the ball. Because one of the most important performance outcomes in spin bowling is spin rate [1], the mechanical efficiency in generating spin will be a prime consideration for spin bowlers in how they apply technique.

Hence, we used the ratio of *ω*_max_ to *T*_*R*max_ as a spin efficiency index (SEI), an index that gives a measure of torque conversion into spin rate. As a rule of thumb, 0.3 Nm generates an average of 30 rps, so that the ratio *ω*_max_/*T*_*R*max_ gives a benchmark value of 100 (reciprocal value of ‘bowling potential’ times 100 [6]). The larger *ω*_max_/*T*_*R*max_, the more efficiently the torque is converted into spin. In addition, we calculated the ratio of *T*_*S*max_ to *T*_*P*max_ as a torque generation index (TEI), rating the bowling deliveries with large *T*_*S*max_ and minimal *T*_*P*max_ as higher performance outcomes.

This study aims to reveal which biomechanical principles apply to the efficient conversion of torque into spin rate for finger-spin and wrist-spin deliveries. Coaches may be able to use these principles to identify and develop spin bowling talent.

## Materials and Methods

### The Smart Cricket Ball

**Fig. 2:**
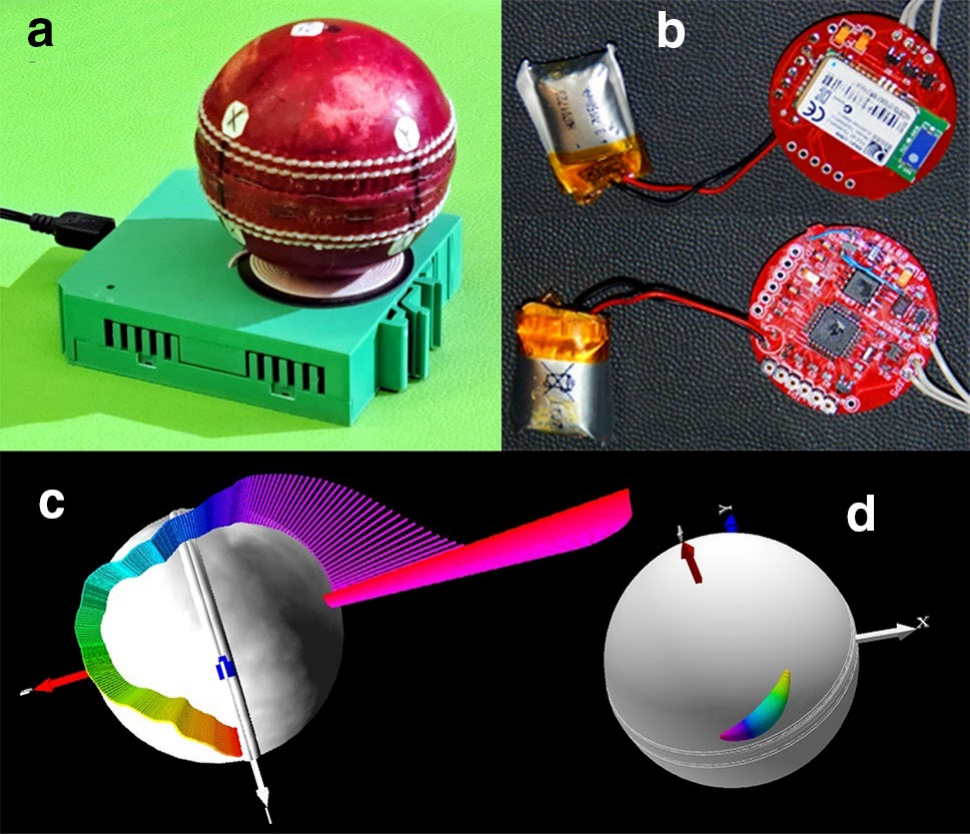
**a** Smart cricket ball on charging dock; **b** electronics; **c** 3D visualization of the vector diagram of the angular velocity (the time is color-coded); **d** 3D visualization of the center of pressure (where the torque is imparted on the ball)

The second prototype of the smart ball (Fig. 2a) was developed in 2014 [7]. It measures the angular velocity with three single-axis high-speed gyroscopes at a frequency of 815 Hz, transmits the data wirelessly to a laptop or smartphone, and is inductively charged [7]. The electronics are miniaturized and have a maximal diameter of 30 mm (Fig. 2b). As with any smart and instrumented sports equipment, the data are processed and visualized [8], especially in 3D (Fig. 2c). In addition to the measured angular velocity *ω*, the software calculates the torques *T*_R_, *T*_S_, and *T*_P_; the angular acceleration *α*, and the power *P* [5]. Furthermore, it computes four newly discovered skill parameters [5]: the precession *p* (speed of the movement of the *ω*-vector, caused by *T*_P_; Fig. 2c), the normalized precession *p*_*n*_ (angle *θ* shown in Fig. 1/inset), the efficiency *η* (ratio of actual to ideal angular kinetic energy, if *θ* and thus *T*_P_ were zero), and the ‘frequency’ (ratio of *α*_max_ to release *ω*_max_). For a sine wave, the latter skill parameter *α*_max_/*ω*_max_ would be 2*πf*, where *f* denotes the frequency of the sine wave. The smaller the ratio *α*_max_/*ω*_max_, the longer the period of the torque-generating cycle and the faster is the generated spin rate relative to angular acceleration and torque *T*_R_. [5]. Note that *α* = *T*_S_/*I* and not *T*_R_/*I*; and *ω* = ∫ *α* d*t*. Even if *ω* is directly calculated from *α*, the peak values of *ω* and *α*, namely *α*_max_ and *ω*_max_, do not correlate perfectly, such that the ratio *α*_max_/*ω*_max_ is not a constant, and rather depends on the time period during which the ball is accelerated. Consequently, bowling deliveries with maximal *ω*_max_ and minimal *α*_max_ are rated as higher performance outcomes.

### Participants

Approvals from corresponding institutional ethics committees were obtained prior to profiling. Between 2012 and 2021, we profiled numerous male spin bowlers (aged 18–48) using the Smart Ball, playing cricket at various levels (from local clubs to first-class cricket). The gender distribution of the participants is explained by the availability and level of performance. Male spin bowlers generally achieve significantly higher standards of performance, including a higher spin rate. All bowlers reported that they were free from injury or dysfunction that would inhibit their bowling performance during the testing. All bowlers were requested to bowl as if under match conditions, whether outside on the oval or inside (indoor gymnasium or 30 m biomechanics lab). The bowlers bowled the ball into a net placed 12 m along the standard pitch length while sighting the target at the wickets.

We used our database of Smart Ball data (412 deliveries) to achieve the above aims. Our database of spin bowlers consists of finger and wrist spinners divided into top, side, and back spinners based on their stock ball. To achieve the above goals, we did not specifically differentiate between finger spinners and wrist spinners as we were looking for the underlying mechanical principles that govern the efficient conversion of torque into spin rate.

### Data Processing

Since the physical performance parameters (*ω*, *T*_R_, *T*_S_, *α*, and *P*) are mathematically related (*ω* = ∫ *α* d*t*; *α* = *T*_S_/*I*; *T*_S_ = *T*_R_ cos*θ*; *P* = *ω T*_S_), and the torque to spin rate conversion is inherently a different skill factor than suggested by Eq. (1), we correlated the skill performance parameters (*T*_P_, *p*, *p*_*n*_, *η*, and *α*_max_/*ω*_max_) with the ratios *ω*_max_/*T*_*R*max_ and *T*_*S*max_/*T*_*P*max_ to identify the greatest influencing factor. We used multiple regressions with five independent variables and two dependent variables, of the general form

2 $$y = a + b_1 x_1 + b_2 x_2 + b_3 x_3 + b_4 x_4 + b_5 x_5 ,$$

where *x*_1–5_ are the independent variables and *y* is the dependent.

The degree of influence of an independent variable on a dependent variable was identified from the standardized regression coefficients and from the increase in multiple *R*^2^ when adding a fifth independent variable to the previous four [9]. The most influential factor was then further examined in terms of its biomechanical implications for optimal or suboptimal torque to spin rate conversion. For all regression analyses, the *R*^2^ and the one-sided *p* value of the trend (positive or negative gradient) were calculated.

## Results

The statistical results of the two multiple regressions are presented in Table 1. In both regressions, the largest contributor to the variance was the ‘frequency’ parameter (*α*_max_/*ω*_max_), followed by the maximum precession torque *T*_*P*max_. Since *T*_*P*max_ is the denominator of the ratio *T*_*S*max_/*T*_*P*max_, which is the TGI, the ideal technique of a spin bowler would be to align the **T**_**R**_ vector with the *ω*-vector and thus to keep *T*_P_ at zero.

**Table 1:** Multiple regressions of skill parameters

Multiple regression of *ω*_max_/*T*_*R*max_ to skill parameters	
*a*	*R*^2^
154.9544	0.6814

*n*	*x*_*n*_	*b*	**|***B***|**	*R*^2^ *w*/*o x*_*n*_	Difference
1	*p*_max_ (rad/s)	− 0.137	0.1497	0.6726	0.0088
2	*θ* (°)	0.0056	0.0109	0.6813	0.0001
3	*T*_*P*max_ (Nm)	− 115.813	0.4314	0.5157	0.1657
4	*η* (%)	0.125	0.1063	0.6747	0.0067
5	*α*_max_/*ω*_max_ (s^–1^)	− 2.4987	0.6574	0.462	0.2194

Multiple regression of *T*_*S*max_/*T*_*P*max_ to skill parameters	
*a*	*R*^2^
− 0.2554	0.8509

*n*	*x*_*n*_	*b*	**|***B***|**	*R*^2^ *w*/*o x*_*n*_	Difference
1	*p*_max_ (rad/s)	− 0.0201	0.4539	0.7698	0.0811
2	*θ* (°)	0.0012	0.0478	0.8492	0.0017
3	*T*_*P*max_ (Nm)	− 6.7202	0.5161	0.6137	0.2372
4	*η* (%)	0.0065	0.1142	0.8432	0.0077
5	*α*_max_/*ω*_max_ (s^–1^)	0.1614	0.8758	0.4616	0.3893

*a*, *b* coefficients of Eq. (2), *B* standardized regression coefficient, *x* independent variables, *n* number of *x*, *R*^2^ multiple coefficient of determination (all 5 *x*), *R*^2^ *w*/*o x*_*n*_ multiple coefficient of determination of 4 independent variables without the variable listed under *x*_*n*_, *difference* increase of ‘*R*^2^ *w*/*o x*_*n*_’ when including the fifth variable (*R*^2^ minus *R*^2^ *w*/*o x*_*n*_), *ω*_max_ angular velocity at release, *T*_*R*max_ peak resultant torque, *T*_*S*max_ peak spin torque, *T*_*P*max_ peak precession torque, *p*_max_ peak precession, *θ* normalized precession (angle between *T*_*R*_ and *ω*), *η* efficiency (ratio of actual to ideal energy), *α*_max_ peak angular acceleration

Subsequently, the SEI (*ω*_max_/*T*_*R*max_) and the TGI (*T*_*S*max_/*T*_*P*max_) were correlated with the frequency parameter *α*_max_/*ω*_max_ with a multiple *R*^2^ of 0.7965, and single *R*^2^ of 0.4687 and 0.4527, respectively. The combined influence, determined from the sum of the single regression *R*^2^ minus the multiple regression *R*^2^, was only 0.1249, which is not surprising, since *ω*_max_/*T*_*R*max_ and *T*_*S*max_/*T*_*P*max_ are indirectly correlated at *R*^2^ of only 0.0246 (still significant, *p* = 0.0007). The individual influences (semi-partial correlations) of *ω*_max_/*T*_*R*max_ and *T*_*S*max_/*T*_*P*max_ on *α*_max_/*ω*_max_, from the single regression *R*^2^ minus the combined influence were 0.3438 and 0.3278, respectively.

**Fig. 3:**
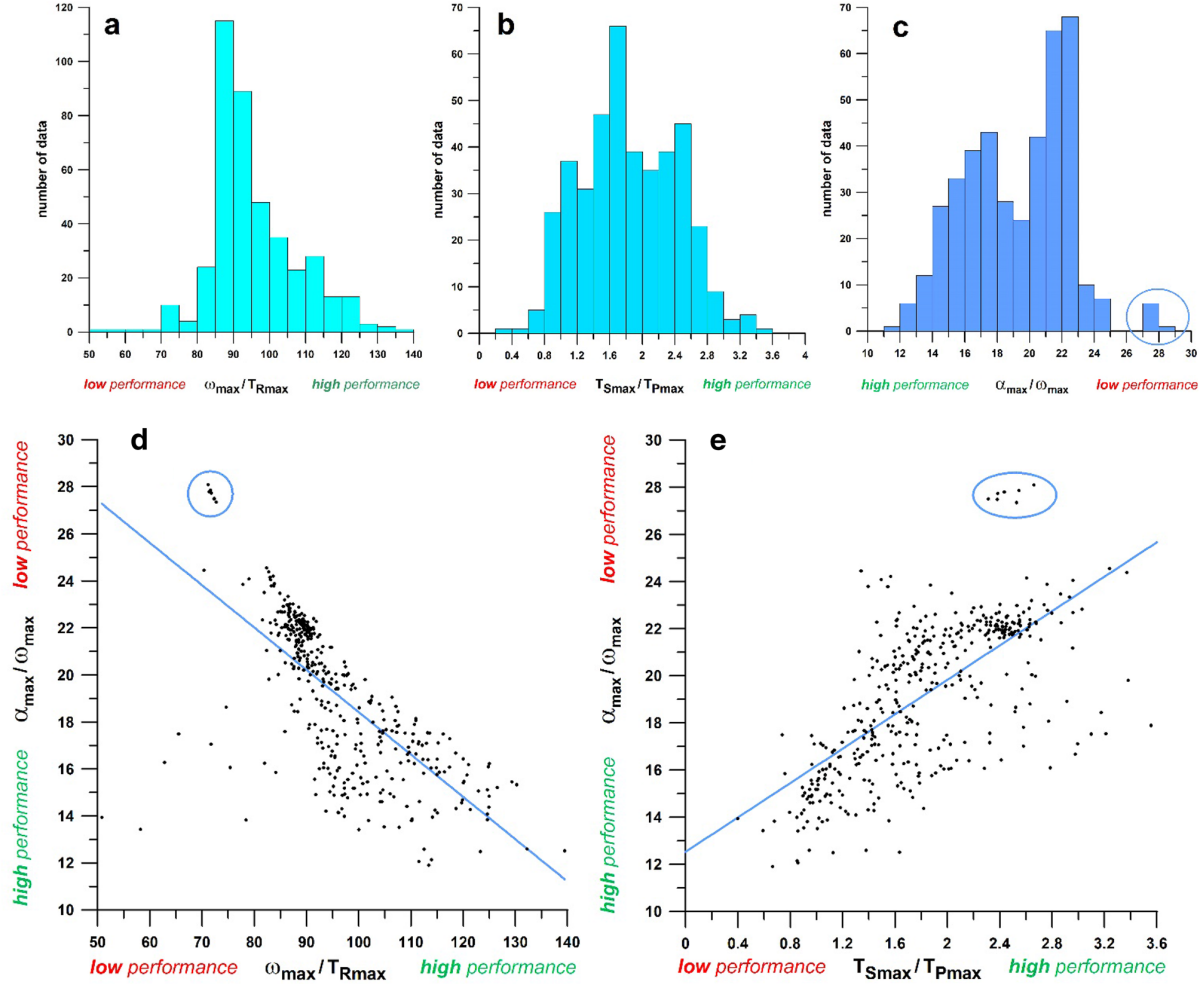
Histograms of *ω*_max_/*T*_*R*max_ (**a**), *T*_*S*max_/*T*_*P*max_ (**b**), and *α*_max_/*ω*_max_ (**c**); and correlations of *α*_max_/*ω*_max_ against *ω*_max_/*T*_*R*max_ (**d**), *T*_*S*max_/*T*_*P*max_ (**e**); the blue circles identify the data of bowler 5

The statistical distributions of the ratios *ω*_max_/*T*_*R*max_, *T*_*S*max_/*T*_*P*max_, and *α*_max_/*ω*_max_ are shown in Fig. 3a–c. In Fig. 3c, the outliers on the right tail of the histogram are from one single bowler.

**Fig. 4:**
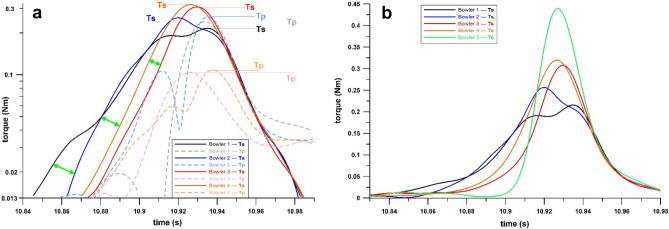
Torque versus time plots aligned, so that the ball release points share the same timestamp; **a** four wrist spinners (bowlers 1–4 in Table 1) and their *T*_S_ and *T*_P_ profiles (the green arrows emphasize the timing of the onset of *T*_S_), **b** *T*_S_ of five bowlers (note that the earlier *T*_S_ is generated, the smaller the peak *T*_S_, but the more torque is converted to spin rate); *T*_S_: spin torque, *T*_P_: precession torque

The correlations of *α*_max_/*ω*_max_ versus *ω*_max_/*T*_*R*max_ and *T*_*S*max_/*T*_*P*max_ are shown in Fig. 3d, e. In Table 1 and Fig. 3d, *α*_max_/*ω*_max_ and *ω*_max_/*T*_*R*max_ are indirectly correlated, which means that high performance values of *α*_max_/*ω*_max_ correspond to high performance values of *ω*_max_/*T*_*R*max_ (since low *α*_max_/*ω*_max_ values are associated with high performance). Conversely, *α*_max_/*ω*_max_ and *T*_*S*max_/*T*_*P*max_ are directly correlated in Table 1 and Fig. 3e, which means that high performance values of *α*_max_/*ω*_max_ correspond to low performance values of *T*_*S*max_/*T*_*P*max_. This is a counterintuitive and unexpected result, interpreted as follows:

If the parameter *α*_max_/*ω*_max_ decreases (increase in performance by increasing *ω*_max_/*T*_*R*max_), the parameter *T*_*S*max_/*T*_*P*max_ decreases (decrease in performance) to values equal to or less than 1, due to increase in *T*_*P*max_ and decrease in *T*_*S*max_. Figure 4a shows the *T*_S_ and *T*_P_ time-series data of four wrist spinners (leg spinners). Table 2 presents the processed data of the four wrist spinners, who have comparable *T*_*R*max_ but slightly different *ω*_max_ (faster for bowlers 1 and 2), and significantly different *ω*_max_/*T*_*R*max_ and *T*_*S*max_/*T*_*P*max_ (better for bowlers 1 and 2). In contrast, bowlers 3 and 4 have excellent *T*_*S*max_ and *T*_*P*max_ data. When calculating the angular impulse Δ*L* of *T*_S_, i.e., Δ*L*_S_, then the data of bowlers 1 and 2 are better, even if their *T*_*S*max_ is lower than that of bowlers 3 and 4. The solution to this paradox is obvious: the time-window *τ* (Table 2), over which the time integral of *T*_S_ is calculated, is wider for bowlers 1 and 2. This phenomenon can be clearly seen in Fig. 4a, as the increase in *T*_S_ starts earlier for bowlers 1 and 2 (Fig. 4a, green double arrows), with the decrease of *T*_S_ occurring simultaneously for all bowlers shortly before releasing the ball. It seems that a wider *T*_S_ time-window *τ* cancels out and even outperforms the effect of the smaller *T*_*S*max_ with respect to the angular impulse Δ*L*_S_. In addition, the time integrals of *T*_R_ and *T*_P_ are also better in bowlers 1 and 2. Hence, a key biomechanical principle for the efficient conversion of torque to spin rate is the angular impulse, which requires the spin bowler to apply torque to the ball well before the release point. However, this practice of increasing the time-window *τ* of torque application seems to come at the expense of smaller *T*_*S*max_. The time-window *τ* correlates with *α*_max_/*ω*_max_ at *R*^2^ of 0.8009.

**Table 2:** Skill parameter values of four wrist spinners

Bowler number (cf. Fig. 4a)	1	2	3	4
*ω*_max_ (rps)	**29.62**	**30.7**	26.91	27.77
*T*_*R*max_ (Nm)	0.3228	0.3333	0.326	0.3238
*T*_*S*max_ (Nm)	0.2158	0.2566	**0.3199**	**0.3079**
*T*_*P*max_ (Nm)	0.2406	0.2614	**0.1082**	**0.1041**
*α*_max_/*ω*_max_ (s^–1^)	**14.91**	**17.11**	21.18	22.69
*ω*_max_/*T*_*R*max_ (rps N^–1^ m^–1^)	**91.74**	**92.09**	82.55	85.77
*T*_*S*max_/*T*_*P*max_ (−)	0.897	0.981	**2.956**	**2.958**
∫ *T*_*R*_ d*t* (Nms)	**0.0185**	**0.0187**	0.0161	0.0148
∫ *T*_*S*_ d*t* = Δ*L*_*S*_ (Nms)	**0.0138**	**0.0137**	0.0119	0.0115
∫ *T*_*P*_ d*t* (Nms)	0.0103	0.0111	**0.0068**	**0.0069**
Time window *τ* (s)	**0.233**	**0.184**	0.164	0.155

High performance data in bold font

*∫* integral symbol, *L*_S_ angular momentum, Δ*L*_S_ angular impulse of spin torque *T*_S_; the time-window *τ* is the integration window of *T*_R_, *T*_S_, and *T*_P_

Considering the outlier data as specific cases, these data were marked with a blue circle in Fig. 3c–e, plotted from torque measurements of Bowler 5 (finger spinner), who generated *T*_*R*max_ values between 0.41 and 0.45 Nm (mean 0.433 Nm) in seven deliveries. These outcomes were only achieved in two out of seven cases (0.41 Nm and 0.42 Nm; mean 0.381 Nm) by Bowler 2 (also finger spinner). However, Bowler 5 showed much worse *α*_max_/*ω*_max_ values ranging from 27.3 to 28.1 s^–1^ (mean, 27.7 s^–1^), compared to the corresponding values of Bowler 2 ranging from 17.6 to 18.7 s^–1^ (mean, 19.4 s^–1^). Accordingly, Bowler’s 5 *ω*_max_/*T*_*R*max_ and *T*_*S*max_/*T*_*P*max_ average ratios were 71.9 (low) and 2.46 (high), respectively (as expected from the paradox explained above); compared to Bowler’s 2 average ratios of 97.5 (high) and 1.47 (low), respectively. The *T*_S_ time data are shown in Fig. 4b, characterized by the delayed onset of *T*_S_ with a short time-window of only 0.130 s. Bowler’s 5 *T*_*R*max_ data ranged between 0.41 Nm and 0.45 Nm, a characteristic of an elite category of wrist spinners, and can be functionally extrapolated to a value of approximately 43 rps; but, at his current level of training, Bowler 5 is unable to convert this level of torque to such a spin rate.

## Discussion

### Mechanics of Spin Bowling and Performance Outcomes

Spin bowling has been an effective form of bowling since the origins of the sport. The skilled spin bowler imparts spin on the ball to cause it to swerve in the air via the Magnus effect and then deviate off the pitch, factors that act to confound the batter’s ability to score runs or prevent a dismissal. In addition, higher spin rates could potentially increase the amount of swerve and lateral deviation. This is consistent with the observation that experienced spin bowlers usually demonstrate elevated mean spin rates in comparison to amateur and non-professional players. Coaches recognize that spin rate is a crucial factor in achieving success in spin bowling, particularly for those seeking to elevate their performance to a professional level. From a biomechanical perspective, this improvement in performance could be achieved by increasing the efficiency of converting the torque applied to the ball into spin rate. The Smart ball is a unique tool that can measure various parameters of spin bowling efficiency. Therefore, in this study, we used the Smart ball to explore the fundamental biomechanical principles that effectively convert torque into spin rate. With this knowledge, coaches could potentially identify and cultivate spin bowling talent by applying these principles.

### Coaching Application: The Time Window

From a practical standpoint, the time frame indicates that angular impulse is a crucial factor in determining the effectiveness of spin production. A spin bowler could increase the angular impulse applied to the ball by increasing the duration of torque application to the ball. One way to improve bowling skills is using specific techniques that focus on increasing the contact time of the fingers on the ball, particularly those using the longest finger [10]. These techniques include utilizing different grips, increasing the range of forearm and wrist motion during the spin torque application, emphasizing spin torque with the middle finger, and making sure that the middle finger is the last to leave the ball's surface [10]. Based on this approach, it is likely that bowlers with longer fingers may have an advantage in applying larger angular impulses due to their longer moment arms and achieving a longer time-window of torque application. Conversely, spin bowlers who apply the spin torque over a relatively shorter time-window would need to increase the magnitude of spin torque as compensation to maintain a similar level of spin rate.

### Advantages of Analyzing Bowling Biomechanics with a Smart Ball

These two biomechanical factors of increasing the torque time-window and reducing precession can only be realistically measured with our Smart Ball, as it does not require cameras and reflective markers like a motion analysis system does and, unlike the Kookaburra Smart Ball (Kookaburra, Melbourne, Australia), has three high-speed gyroscopes embedded in the ball.

Working on the premise that elite spin bowlers are the most skillful at imparting spin to the ball, we correlated the skill performance parameters (*T*_P_, *p*, *p*_*n*_, *η*, *α*_max_/*ω*_max_) with the SEI (*ω*_max_/*T*_*R*max_) and the TGI (*T*_*S*max_/*T*_*P*max_). In both the regressions, the largest contributor to the variance was ‘frequency’ parameter (*α*_max_/*ω*_max_) followed by the maximum precession torque, *T*_*P*max_. The frequency parameter indicates that the underlying mechanism for converting torque *T*_R_ into spin rate *ω* is an early onset of *T*_S_ that allows a wide time-window *τ* over which *T*_S_ can be generated. An intuitive way of understanding the frequency parameter is to consider the units (s^−1^). Now, if *T*_S_ were a sine wave, the skill parameter *α*_max_/*ω*_max_ would be 2*πf*, where the variable *f* denotes the frequency of the sine wave. The reciprocal of *f*, i.e., 1/*f*, is identical to the duration of a single sine-wave cycle and is proportional to the time-window *τ*. Both the time-window and its substitute *α*_max_/*ω*_max_ are therefore very useful for performance profiling and talent identification. This distinguishes our Smart Ball from the Kookaburra Smart Ball (Kookaburra, Melbourne, Australia), which only calculates one spin rate data value in the early phase of the ball's flight. The magnetometer data's oscillation frequency appears to be the underlying source of the spin rate information [11], since commercially available low-speed gyroscopes possess the capability to measure spin rates only up to 5.5 rps. Only one specific gyroscope (InvenSense, San Jose, California, USA) can measure up to 11 rps. In addition, a single spin rate data value does not allow calculation of further physical performance parameters (continuous *ω*, *α*, *T*_R_, *T*_S_, and *P*), let alone skill parameters (*p*, *θ*, *T*_P_, *η*, *α*_max_/*ω*_max_). Furthermore, assessing spin bowling performance solely on a single spin rate data value can be misleading, because the spin rate is not only affected by performance, but also by the type of delivery. For instance, topspin deliveries, except for the Googly, produce a higher spin rate than backspin. Similarly, wrist spin deliveries generate more spin rate than finger spin [5]. Choosing young bowlers based on just one spin rate data value would only benefit wrist spin top spinners.

### Reassessment of Skill Parameters

In fast bowling, the connection between spin rate and the plane of bowling shoulder circumduction is evident. It has been well established that fast bowlers tend to convert torque to spin rate inefficiently, when the SEI (*ω*_max_/*T*_*R*max_) is less than 70, and when the frequency parameter (*α*_max_/*ω*_max_) is greater than 25 [12, 13]. When a bowler delivers the ball, the direction of rotation of the bowling arm holding the ball corresponds to topspin. However, the ball is released with a backspin, which causes the angular velocity vector to turn 180° from topspin to backspin. Consequently, there is a decrease in the angular velocity at this transition [12, 13].

Given the usefulness of the skill parameter *α*_max_/*ω*_max_ and its connection to converting *T*_R_ into *ω*, the list of skill parameters (*p*, *θ*, *T*_P_, *η*, *α*_max_/*ω*_max_) needs to be revisited. The precession torque *T*_P_ seems to have lost its importance, since it cannot be kept to a minimum. On the contrary, it is surpassed and even counteracted by early *T*_S_ onset, which decreases *T*_*S*max_ and increases *T*_*P*max_. The normalized precession is still useful for other purposes, namely to assess the angle *θ* (Fig. 1) in the early stages of torque production. Different spin bowling deliveries show clear differences in the skill parameters *p*_max_, *θ*, *η*, and *α*_max_/*ω*_max_ [5]. The improvement in skill parameters as an effect of the training intervention showed that *θ* and *η* improved significantly, while *p*_max_, *T*_*S*max_/*T*_*P*max_, and *α*_max_/*ω*_max_ did not change at all.

Comparing the time windows *τ* of bowlers 1 and 5, 0.233 s and 0.130 s, respectively, then bowler 1 produces the onset of *T*_S_ 0.103 s earlier than bowler 5. Although the exact mechanism responsible for early *T*_S_ onset is unknown, this study provides evidence that the conversion of the resultant torque *T*_R_ (the total torque the bowler produces) into spin rate depends on how early the spin torque *T*_S_ is generated relative to the release point. The earlier generation of spin torque corresponds with a longer overall time-window, which increases the angular impulse for spin generation, despite paradoxically suffering from a decreased magnitude.

Our study has demonstrated that the smart cricket ball represents a novel and effective tool for analyzing the mechanics of spin bowling. Through its capacity to evaluate multiple skill parameters, spin bowlers can be evaluated on their ability to efficiently convert spin torque into spin rate. Our research findings have revealed that the width of the torque window is associated with spin bowling effectiveness, with the spin angular impulse serving as the underlying principle for this efficiency. These results have significant implications for coaching and players alike. Techniques aimed at optimizing the spin angular impulse for spin bowlers could be explored, particularly given that these players, particularly wrist spinners, are a vital weapon in cricket across all forms of the game, including the lucrative T20 league. The smart ball could be used to provide bowlers with intelligent feedback that can help them improve their skills and excel at the highest levels, thereby increasing the potential for cricket teams to achieve success.

## Data Availability

The raw data supporting the conclusions of this article will be made available by the authors to any qualified researcher, if they have obtained Ethics Approval for secondary use of existing data through a Consent Waiver.
